# Supplementary material for: The Striking Flower-in-Flower Phenotype of Arabidopsis thaliana Nossen (No-0) is Caused by a Novel LEAFY Allele
Source: Plants (Basel). 2019 Dec 13;8(12):599. doi: 10.3390/plants8120599 (PMC6963406; doi:10.3390/plants8120599)
Supplement: Supplementary file 1 [file plants-08-00599-s001.pdf]

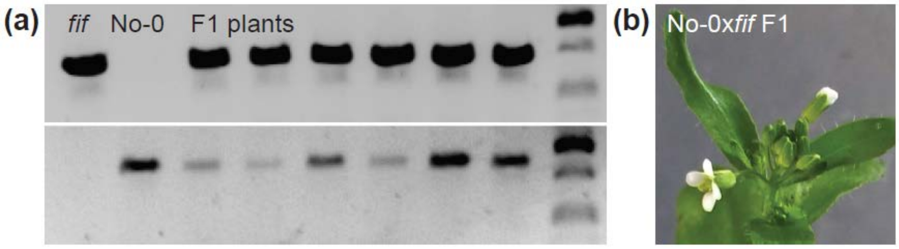

Figure S1. Proof of the successful backcross of the *fif* mutant with wild type No-0. **(a)** Genotyping PCR analysis of F1 plant material using a primer pair (sense: 5´-CGTGAAACTCAAGGCATTCTCTACTTC -3´; antisense: 5´-CGATTTCGACTTTAACCCGACCGG -3´) specifically amplifying the Ds transposon (upper row) and a primer pair (sense: see above; antisense: 5´-CGTACGTAGAACAACAGAGAATAAGC-3´) specifically amplifying the wild-type genomic region (lower row). **(b)** Representative image of the inflorescence and flower phenotype of F1 plants generated by the cross of the *fif* mutant with wild type No-0 (♀No-0 x ♂*fif*). The reciprocal cross (♀*fif* x ♂No-0) provided identical results.

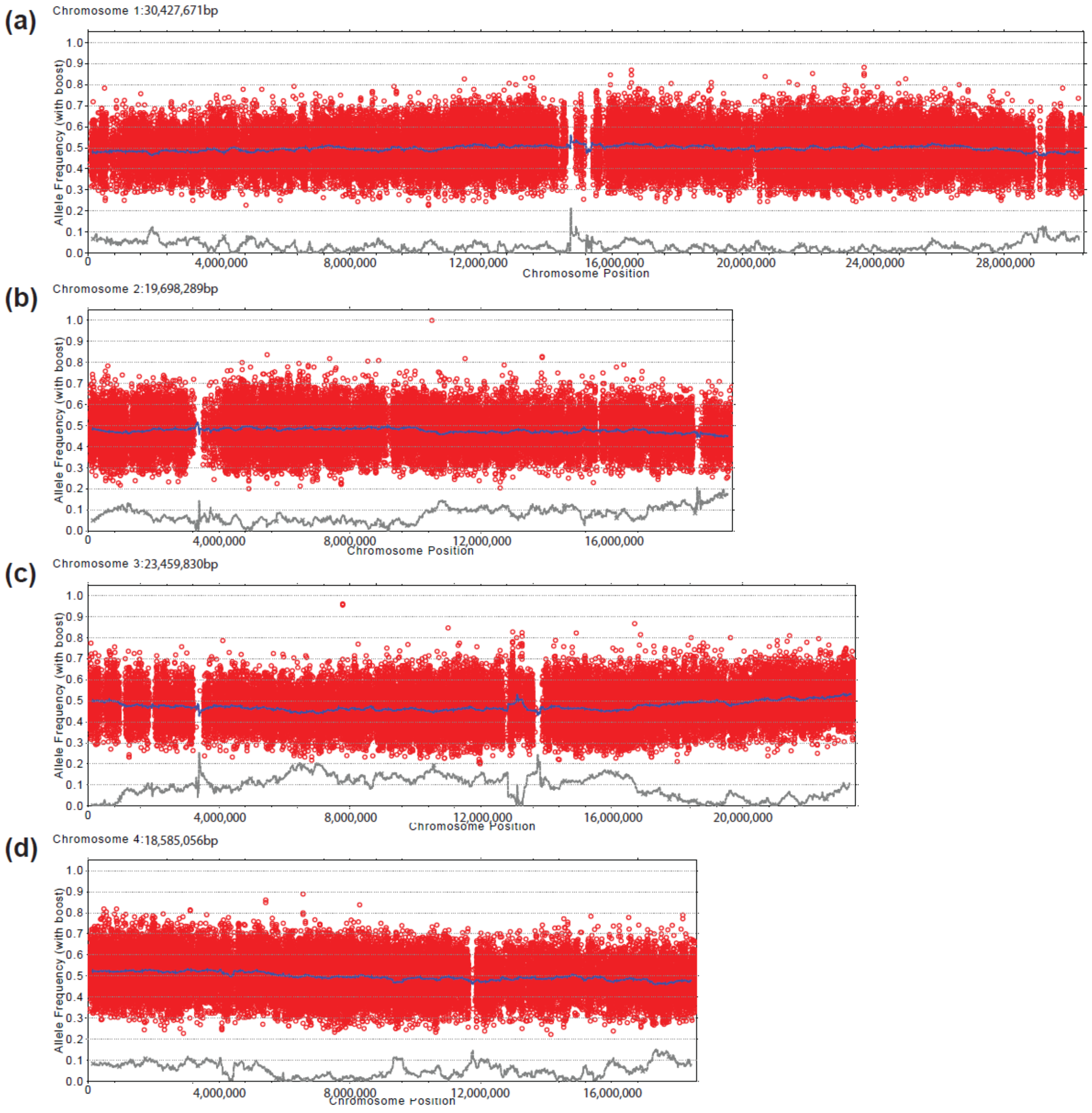

Figure S2. Allele frequency analysis of the No-0 allele within the recombinant mutant pool (unlinked chromosomes). Each red circle refers to a SNP marker distinguishing the No-0 and Col-0 genotypes. The blue line refers to a 200 kb sliding window analysis of the allele frequencies. The brown line would highlight potential mapping intervals (x-axis: genomic location; y-axis: No-0 allele frequency). **(a)** Chromosome 1. **(b)** Chromosome 2. **(c)** Chromosome 3. **(d)** Chromosome 4.

Name		Sequence	Marker Type		bp *Col-0*	bp *No-0*
7_1-8660	S	GCGGCACAACCTAAATGAAA	INDEL		189	168
	A	TGCATGCAATTATCACGTATG				
15_1-26627	S	GCAATTCATCAGCAGGAGGT	INDEL		245	261
	A	ATCAGGGAGCAAAATGCAAG				
20_2-12717	S	AAAATGGGGCCTAATACGTTG	INDEL		403	~180
	A	CAAAGGAAACACCTGCATCA				
4_3-3716	S	TAATGGTGGCCCAATCTCAT	INDEL		1482	613
	A	AATTCCAAATGGAGCCACAA				
16_3-20726	S	GGGCCCATTTCAACTAAGGA	INDEL		149	~160
	A	TCTCACAAGCCCAGTAAAAACT				
42_4-17544	S	CACCATTGACATTTGATGCAC	INDEL		214	234
	A	CCGTAGCTCCATTGGCTTAT				
1_5-1576	S	CAGCTCCGACGATGATGATA	INDEL		363	~420
	A	TGGAGTAATTGTTCCTTCACAAA				
37_5-22317	S	GCATTGAAATAGTGTTTTTAACCAAA	INDEL		132	152
	A	TGTTGGTTGCCACCTTATCA				
19_5-17388	S	TTTTGCAAGTCCGTAGTCAATG	INDEL		110	121
	A	TTTGGTTTTGGAATTTCTTTTG				
35_5-19138	S	AACTCATGCAATGCGACATC	INDEL		182	164
	A	CCCGTCCATGATCTGTTTCT				
37_5-22317	S	GCATTGAAATAGTGTTTTTAACCAAA	INDEL		132	152
	A	TGTTGGTTGCCACCTTATCA				
				Enzyme		
S5-16	S	CACGAGAGATACCTGCAAAACAG	dCAP	DraII	160	134+26
	A	CAAACGCTTTTGAAATCATGGGT**C**C				
S5-24	S	GTAATACACAACAATGGGGAG	dCAP	Esp3I	244+43+26	287+26
	A	CATATTCGAGTTCTGATGCACAC				
5-LFY	S	TATCTGTTCCACTTGTACGAA**GT**AT	dCAP	AccI	150	129+21
	A	CATAAATTTCAAGATAATGAACGGTC				
5-LFY#2	S	TATCTGTTCCACTTGTACGAA**GC**AT	dCAP	SphI	129+21	150
	A	Same as 5-LFY				

Table S1 Names and sequences of the INDEL and dCAP primers. Bold and underlined: introduced mismatches to incorporate an ecotype specific restriction site in the PCR product

Name			sequence
pAP1	S	Bio-	aaaaaGAAGGACCAGTGGTCCGTACaaaaa
	A		tttttGTACGGACCACTGGTCCTTCttttt
pAP1m	S	Bio-	aaaaaGAAGGAAAAGTAATCCGTACaaaaa
	A		tttttGTACGGATTACTTTTCCTTCttttt
C28M12	S	Bio-	aaaaaaTTTATACTTGATCATaaCTTaaaa
	A		ttttAAGttATGATCAAGTATAAAtttttt

Table S2 Sequences of the dsDNA oligonucleotides used in the DPI-ELISA.
